# Combining immune checkpoints with TNFSF agonists: a new horizon for cancer and autoimmune therapies

**DOI:** 10.3389/fimmu.2025.1557176

**Published:** 2025-03-17

**Authors:** Lele Sun, Cuiping Li, Tingting Gao, Zhe Liu, Yanli Hou, Wei Han

**Affiliations:** ^1^ Department of Clinical Pharmacy, Zibo Central Hospital, Zibo, Shandong, China; ^2^ Health Management Center, Zibo Central Hospital, Zibo, Shandong, China; ^3^ Department of Hepatobiliary Surgery, Zibo Central Hospital, Zibo, Shandong, China; ^4^ Department of Pharmacy, Zibo First Hospital, Zibo, Shandong, China; ^5^ Traumatic Orthopaedic Ward, Zibo Central Hospital, Zibo, Shandong, China; ^6^ Hematology Department, Zibo Central Hospital, Zibo, Shandong, China

**Keywords:** immune checkpoints, TNFSF agonists, cancer therapy, autoimmune diseases, immune response, biomarkers

## 1 Introduction

**Figure 1:**
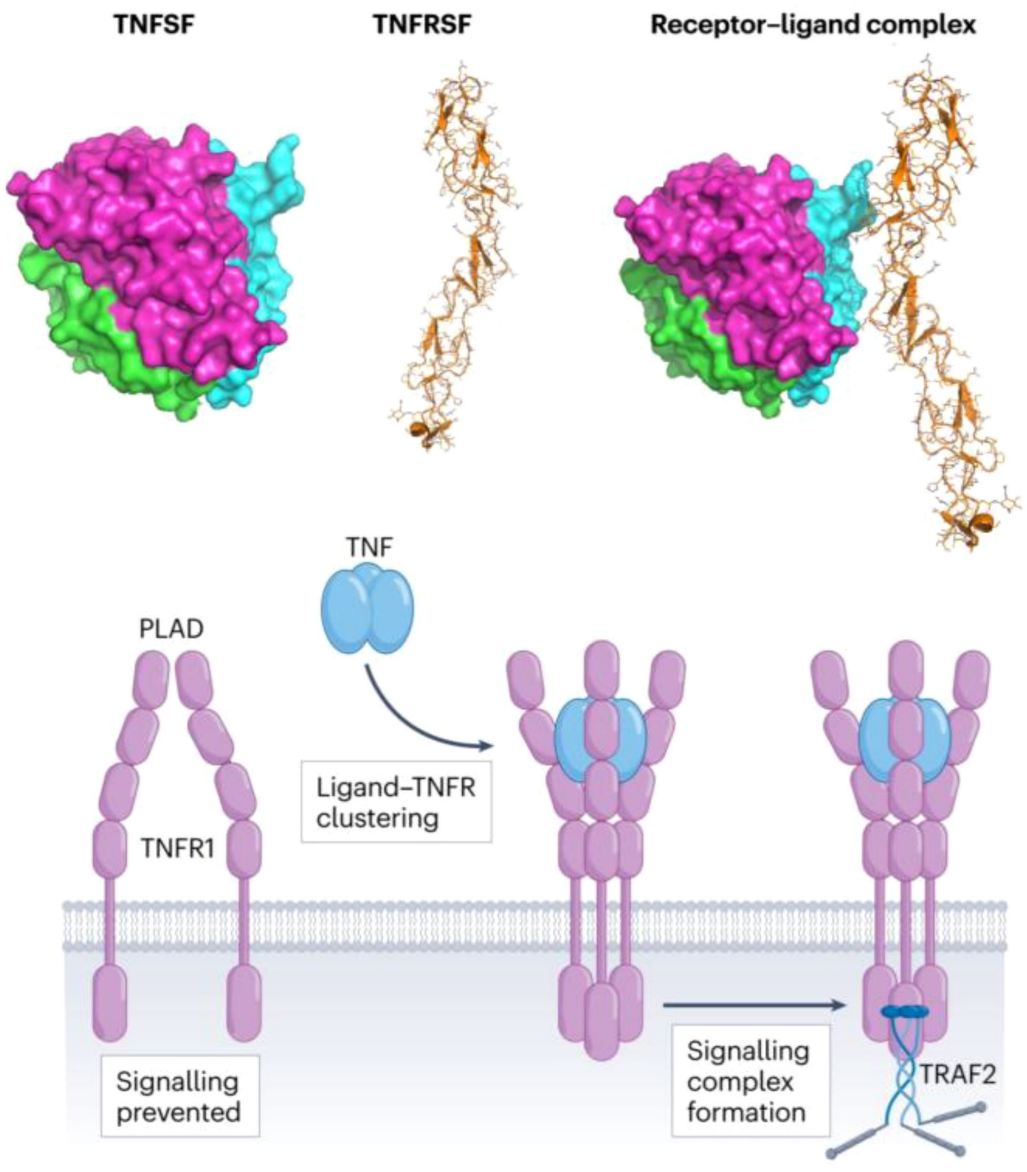
TNFSF and receptor-ligand complex formation. The structure of TNFSF (light purple) in its unbound state (left), followed by its binding to the TNFSF receptor (green, blue, and pink) (middle). The right panel shows the receptor-ligand complex, with the TNFSF (orange) bound to the receptor, initiating the formation of the signaling complex. The lower part illustrates how ligand-receptor clustering triggers signaling activation, leading to the formation of the TRAF2 signaling complex, essential for modulating immune responses.

The immune response is orchestrated by a complex network of cytokines, which play a fundamental role in both autoimmune diseases and cancer therapy. Immune checkpoint inhibitors (ICIs), which target key inhibitory pathways such as PD-1/PD-L1 and CTLA-4, have revolutionized cancer immunotherapy by reinstating T-cell activity and overcoming immune evasion mechanisms (1). Despite their success in cancers like melanoma and lung cancer, a substantial number of patients fail to respond to ICIs. This resistance is often due to the lack of pre-existing T-cell immunity, the emergence of immune resistance mechanisms, or an immunosuppressive tumor microenvironment (TME), highlighting the need for more targeted therapeutic strategies (2). In this context, cytokines and their receptor networks, particularly those within the tumor necrosis factor superfamily (TNFSF) and its receptors (TNFRSF), have emerged as pivotal players in regulating immune responses. **Figure 1** illustrates the TNFSF signaling pathway. It shows the structure of TNFSF in its unbound state and its interaction with the TNFSF receptor. Upon binding, the TNFSF receptor forms a receptor-ligand complex, as depicted in the middle portion. The lower part highlights the downstream signaling events following receptor-ligand clustering. This clustering activates the TRAF2 signaling complex, which plays a critical role in modulating immune responses. These interactions are fundamental to immune regulation and enhance the therapeutic potential of targeting TNFSF pathways (3). Cytokines such as OX40, 4-1BB, and CD40 promote T-cell activation, survival, and proliferation, contributing to both anti-tumor immunity and immune homeostasis (3). However, their therapeutic potential is often constrained by T-cell exhaustion and the immunosuppressive effects of the TME, which can limit their efficacy, similarly to ICIs (4).

To overcome these limitations, combining ICIs with TNFSF agonists presents a synergistic strategy. While ICIs unmask immune suppression and restore T-cell responses, TNFSF agonists, like OX40 and 4-1BB, can enhance T-cell activation and proliferation, providing a more robust immune response (5). Furthermore, TNFR2 agonists could modulate immune regulation, particularly by influencing regulatory T-cell activity, offering potential benefits for autoimmune diseases.

However, the integration of ICIs and TNFSF agonists also faces challenges, such as immune-related adverse events (irAEs), optimal dosing strategies, and identifying patients who would benefit the most from these therapies (6). Precision medicine, guided by biomarkers and advanced diagnostic techniques, will be critical in overcoming these hurdles. This *Opinion Article* explores how combining ICIs with TNFSF agonists could offer a novel approach for modulating cytokine networks and advancing treatment outcomes in both autoimmune diseases and cancer.

## 2 The complementary mechanisms of ICIs and TNFSF agonists

ICIs have revolutionized cancer therapy by targeting inhibitory pathways such as PD-1, PD-L1, and CTLA-4, restoring T-cell activity and enhancing tumor elimination. However, ICIs often fail in patients with low T-cell infiltration or TME, emphasizing the need for complementary strategies (7). TNFSF agonists, targeting receptors like OX40, 4-1BB, and CD40, provide such a complement by amplifying T-cell activation and proliferation through co-stimulatory signals (8). OX40 agonists enhance CD4+ and CD8+ T-cell responses, while 4-1BB agonists promote CD8+ T-cell cytotoxicity and natural killer (NK) cell activity. Similarly, CD40 agonists stimulate antigen-presenting cells (APCs), fostering robust adaptive immunity. These agonists can reduce T-cell exhaustion and counteract the immunosuppressive influences of regulatory T cells (Tregs) and myeloid-derived suppressor cells (MDSCs) within the TME. **Table 1** summarizes key TNFSF agonists in clinical development, including Urelumab and Selicrelumab for cancer and ABBV-927 for autoimmune diseases. These agents highlight the diverse applications and potential of TNFSF agonists in combination therapies. However, combining these potent immunotherapies raises concerns about immune-related adverse events (irAEs), such as cytokine release syndrome and autoimmunity. Careful patient selection, biomarker-driven stratification, and optimized dosing strategies are essential to mitigate these risks.

**Table 1:** Summary of TNFSF agonists and their therapeutic applications.

Drug Name	Target Receptor	Mechanism of Action	Potential Applications	Current Development Stage	Ref
Rocatinlimab (KHK4083)	OX40 (TNFRSF4)	Enhances CD4+ and CD8+ T-cell activation, cytokine production, and memory formation	Cancer (solid tumors), atopic dermatitis	Phase II/III clinical trials	(9)
Urelumab (BMS-663513)	4-1BB (TNFRSF9)	Promotes CD8+ T-cell proliferation, survival, and cytotoxicity; activates NK cells	Cancer (solid tumors, hematologic malignancies)	Phase I/II clinical trials	(10)
AGEN2373	4-1BB (TNFRSF9)	Selectively activates 4-1BB without causing systemic toxicity	Cancer (solid tumors)	Phase I trials	(11)
Selicrelumab (RG7876)	CD40 (TNFRSF5)	Activates antigen-presenting cells (APCs), enhancing T-cell priming	Cancer (solid tumors)	Phase I clinical trials	(12)
MEDI5083	CD40 (TNFRSF5)	Promotes T-cell activation and dendritic cell maturation	Cancer (solid tumors)	Preclinical trials	(13)
ABBV-927	TNFR2 (TNFRSF1B)	Promotes Treg expansion and suppresses inflammatory immune responses	Autoimmune diseases (e.g., rheumatoid arthritis)	Preclinical trials	(14)
Varlilumab (CDX-1127)	CD27 (TNFRSF7)	Enhances T-cell activation, survival, and memory formation	Cancer (hematologic malignancies)	Phase II clinical trials	(15)
PF-06480605 (RVT-3101)	TL1A (TNFSF15/DR3)	Modulates effector T cells and inflammatory pathways in autoimmune diseases	Ulcerative colitis, Crohn’s disease	Phase II/III clinical trials	(16)
CERC-002	LIGHT (TNFSF14)	Activates HVEM and LTβR pathways, promoting lymphoid organization and inflammation	Cancer, inflammatory disorders	Phase II clinical trials	(17)

The synergy between ICIs and TNFSF agonists arises from their complementary mechanisms. ICIs unmask suppressed T-cell activity, creating a fertile ground for TNFSF agonists to amplify immune responses. Preclinical and early clinical data demonstrate that combinations like PD-1 blockade with 4-1BB or OX40 agonists significantly enhance T-cell infiltration, tumor regression, and survival.

## 3 Advances and challenges in cancer therapy

### 3.1 Advances in the combination approach

Recent preclinical and clinical studies have highlighted the potential benefits of combining ICIs with TNFSF agonists. TNFSF agonists activate immune effector functions by enhancing T-cell proliferation, survival, and cytokine production while reducing T-cell exhaustion. For example, OX40 agonists promote CD4+ and CD8+ T-cell responses and facilitate the generation of memory T cells, which are crucial for long-term anti-tumor immunity. Similarly, 4-1BB agonists increase the cytotoxic capacity of CD8+ T cells and natural killer (NK) cells, amplifying their ability to target tumor cells. CD40 agonists, on the other hand, enhance the antigen-presenting capacity of dendritic cells and macrophages, promoting the activation of tumor-specific T cells (18).

When combined with ICIs, these effects are further magnified. ICIs release the brakes on T-cell activity by blocking inhibitory pathways like PD-1/PD-L1 and CTLA-4, allowing TNFSF agonists to act as accelerators that drive immune responses to a higher magnitude. Preclinical models have demonstrated that the combination of PD-1 blockade and 4-1BB agonists significantly improves tumor infiltration by effector T cells and reduces the suppressive influence of regulatory Tregs and myeloid-derived suppressor cells (MDSCs). Similarly, combining OX40 agonists with CTLA-4 inhibitors has shown superior anti-tumor efficacy compared to either agent alone, underscoring the synergistic potential of these therapies (19, 20). The combination of OX40 agonists and CTLA-4 inhibitors has shown both positive and inconclusive results in clinical trials. In preclinical studies, it promotes robust anti-tumor activity and tumor-free survival. However, in some clinical trials, the combination therapies with ipilimumab (a CTLA-4 inhibitor) and OX40 agonists like BMS-986178 did not improve the clinical response rate compared to monotherapy. The safety profiles of these combinations varied, with common side effects including lymphopenia, rash, pyrexia, and fatigue. Further research is needed to optimize this combination for better clinical outcomes (21).

Clinical trials have started to explore these combinations in various cancers, including melanoma, non-small cell lung cancer, and colorectal cancer. Early-phase trials of 4-1BB agonists with PD-1 inhibitors have shown promising results, with improved objective response rates and progression-free survival (22–24). Furthermore, the combination of PD-1 blockade and 4-1BB agonists shows a synergistic survival benefit in a CD8^+^ T-cell-dependent manner in murine gliomas. It reduces TIL exhaustion and improves TIL functionality. Efficacy correlates with 4-1BB expression on CD8^+^ TILs, not with tumor location or histology, and can license combination therapy in models where TIL 4-1BB levels were previously low (25). OX40 agonists are also being investigated in combination with ICIs, with preliminary data suggesting enhanced tumor regression and durable responses (26–28). Furthermore, CD40 agonists, when paired with ICIs, have demonstrated the ability to convert immunologically cold tumors into hot ones, rendering them more susceptible to immune attack (29, 30). In an ongoing Phase I/II study, the combination of intratumoral sotigalimab (CD40 agonist) and systemic pembrolizumab (anti-PD-1) was evaluated in treatment-naïve, unresectable stage III/IV metastatic melanoma patients. As of December 2021, 30 participants were enrolled, and the combination was well-tolerated with no treatment-related discontinuations or deaths. The most common adverse events were injection-site reactions, with 6 patients experiencing grade-3 immune-related adverse events. The overall response rate (ORR) was 50% (5 complete responses, 10 partial responses), with a disease control rate of 67%. Notably, responses were observed in PD-L1 negative patients and those with elevated LDH. Immunologic analyses showed increased immune activation, including higher T cell clonality and gene expression related to antigen presentation, correlating with clinical responses. This combination therapy demonstrated promising efficacy and immune activation, warranting further investigation (31).

### 3.2 Challenges in implementation

Despite the encouraging progress, significant challenges remain in realizing the full potential of ICI and TNFSF agonist combinations. One of the primary concerns is the risk of irAEs. Both ICIs and TNFSF agonists can independently induce immune activation-related toxicities, such as cytokine release syndrome (CRS), autoimmunity, and systemic inflammation. When combined, these therapies may exacerbate such adverse effects, necessitating careful dose optimization and patient monitoring (32).

Another challenge lies in target specificity. TNFSF receptors are often expressed on multiple cell types, including Tregs and activated T cells, leading to potential off-target effects. For instance, while TNFR2 agonists can enhance Treg function in autoimmune settings, their role in the TME may be context-dependent, potentially favoring either immune suppression or activation. Similarly, OX40 and 4-1BB agonists may inadvertently activate pathogenic T-cell subsets, complicating therapeutic outcomes. Patient stratification and biomarker identification also pose significant hurdles. The heterogeneity of immune responses across different cancers and patient populations requires a precision medicine approach to identify those most likely to benefit from these combinations. Biomarkers such as PD-L1 expression, T-cell infiltration, and TNFSF receptor expression levels could guide patient selection, but their predictive value needs further validation in clinical trials (33).

Finally, logistical challenges in trial design and execution must be addressed (34). Combining ICIs and TNFSF agonists requires careful consideration of dosing schedules, administration sequences, and combination partners to maximize efficacy while minimizing toxicity. Developing standardized protocols and establishing robust clinical endpoints are critical to accelerating the translation of these therapies into practice.

## 4 Potential applications in autoimmune diseases

The combination of ICIs and TNFSF agonists offers significant opportunities in the treatment of autoimmune diseases, addressing the delicate balance between immune activation and suppression. ICIs, such as PD-1/PD-L1 inhibitors, have shown potential in modulating immune responses in autoimmune contexts, but their broad activation of T cells often leads to the risk of exacerbating autoimmunity (35, 36). TNFSF agonists, by contrast, provide a more targeted approach, leveraging their co-stimulatory or regulatory properties to fine-tune immune responses (37).

For example, TNFR2 agonists have emerged as promising candidates for promoting Treg expansion, which is critical in maintaining immune tolerance and mitigating hyperactive immune responses (38). This mechanism is particularly relevant in diseases like rheumatoid arthritis, where inflammation driven by effector T cells and myeloid pathways is predominant. Studies have demonstrated that TNFR2 activation not only enhances Treg function but also reduces the activity of inflammatory cells such as macrophages and dendritic cells. Additionally, OX40 and DR3 agonists have shown potential in selectively targeting pathogenic T-cell subsets without overactivating the entire immune system. This specificity is especially valuable in chronic autoimmune conditions like lupus and multiple sclerosis, where the immune system is persistently hyperactive. By restoring immune homeostasis, these therapies could reduce disease flares while minimizing the risk of global immunosuppression (38, 39). Furthermore, in systemic lupus erythematosus (SLE), Anifrolumab has shown positive results in Phase II and III clinical trials. In August 2021, the drug was approved by the FDA for the treatment of moderate to severe active SLE. Clinical trial results demonstrated significant efficacy in improving disease activity and organ-specific markers, particularly in patients with elevated interferon-I levels (40, 41).

The complementary mechanisms of ICIs and TNFSF agonists create unique therapeutic synergies. ICIs can reactivate suppressed immune responses, while TNFSF agonists enhance the regulation and functionality of these responses. For instance, ICIs could reverse T-cell exhaustion in autoimmune diseases with underlying viral infections, while TNFSF agonists like TNFR2 or CD40 could enhance the tolerogenic capacity of immune cells. Furthermore, combining these therapies with existing treatments such as biologics or small-molecule inhibitors opens new avenues for improving outcomes. Targeted use of such combinations has the potential to achieve a more durable remission in diseases traditionally refractory to single-agent therapies, representing a significant advancement in the field of autoimmune therapeutics (42).

## 5 Future directions

The combination of ICIs and TNFSF agonists represents a rapidly evolving area of immunotherapy with vast potential for addressing unmet needs in cancer and autoimmune diseases. However, to fully realize this potential, several key areas demand focused research and innovation.

### 5.1 Development of next-generation TNFSF Agonists

While first-generation TNFSF agonists have shown promise, their broad activity on multiple cell types poses challenges, including off-target effects and systemic toxicity. Next-generation TNFSF agonists should focus on achieving higher target specificity and controllable activation (43). For example, a recent study demonstrated the development of a bispecific OX40–PD-L1 antibody, which selectively targets tumors while minimizing off-target effects (9, 44, 45). Advances in antibody engineering, such as bispecific antibodies or conditional agonists, could allow for more precise delivery of TNFSF signals to specific immune cells within the tumor microenvironment (TME) or sites of autoimmune pathology. For example, designing agonists that activate only in the presence of high levels of PD-L1 or other markers characteristic of tumors could reduce systemic activation and associated toxicities.

### 5.2 Precision medicine and biomarker-driven therapies

One of the most critical challenges in combinatory immunotherapy is identifying the right patient populations that will benefit from ICIs and TNFSF agonists. Biomarker development is paramount for patient stratification and therapy optimization (46). A study by Zhang et al. (2022) highlighted how TNFSF receptor expression levels can predict the efficacy of TNFSF agonist therapies, especially in patients with high immune infiltration (35). For instance, biomarkers such as PD-L1 expression, immune cell infiltration (e.g., CD8+ T cells), or TNFRSF receptor density could guide patient selection and predict therapeutic responses. Advances in high-throughput sequencing, single-cell analysis, and artificial intelligence could further enhance our ability to identify predictive and prognostic biomarkers, paving the way for truly personalized immunotherapy.

### 5.3 Combining with other therapeutic modalities

While ICIs and TNFSF agonists offer complementary benefits, their efficacy may be further amplified when combined with other therapeutic modalities (47). Such as **Traditional Therapies**: Combining these agents with chemotherapy or radiotherapy could enhance tumor antigen presentation, creating a more immunogenic TME. **Targeted Therapies**: Small-molecule inhibitors targeting oncogenic pathways could synergize with ICIs and TNFSF agonists by reducing immunosuppressive signaling in tumors. **Vaccines and Oncolytic Viruses**: Immunotherapy combinations with tumor vaccines or oncolytic viruses may further boost immune activation and generate robust memory T-cell responses. These multimodal approaches could significantly enhance therapeutic outcomes, particularly in refractory cancers or advanced autoimmune diseases. Combining these agents with chemotherapy or radiotherapy could enhance tumor antigen presentation, creating a more immunogenic TME (20).

### 5.4 Expanding applications beyond cancer

While the focus of current research is predominantly on cancer therapy, the combination of ICIs and TNFSF agonists holds untapped potential in autoimmune diseases. TNFR2 agonists, for example, could enhance Treg function to counteract immune hyperactivation, while OX40 and DR3 agonists could selectively modulate pathogenic T-cell responses. Expanding clinical investigations to include autoimmune indications such as rheumatoid arthritis, lupus, and multiple sclerosis could unlock new therapeutic avenues. For instance, a Phase II trial evaluating the combination of PD-1 inhibitors and OX40 agonists in lupus patients demonstrated promising results, with increased remission rates (36).

### 5.5 Enhancing preclinical and clinical trial design

Robust preclinical models that accurately recapitulate human disease are essential for predicting therapeutic efficacy and toxicity (48). Advances in organoid systems, humanized mouse models, and computational simulations could improve the translation of preclinical findings into clinical success. Recent trials have shown that personalized treatment regimens, guided by biomarkers such as immune checkpoint expression, can significantly improve patient outcomes (19). On the clinical front, adaptive trial designs incorporating biomarker-driven stratification and real-time monitoring could accelerate the development of effective combination therapies.

### 5.6 Cross-disciplinary collaboration

The complexity of combining ICIs and TNFSF agonists necessitates collaboration across disciplines (49). Immunologists, oncologists, computational biologists, and pharmaceutical scientists must work together to integrate insights from basic research, clinical practice, and technological innovation. Partnerships between academia, industry, and regulatory agencies will also be crucial in streamlining the pathway from discovery to clinical implementation. Successful examples of cross-disciplinary collaboration include the development of combination therapies that have progressed through both preclinical and clinical stages (22).

## 6 Conclusion

The integration of ICIs and TNFSF agonists provides a promising approach to modulating cytokine networks, enhancing immune responses in both cancer and autoimmune diseases. ICIs work by lifting immune suppression, allowing for the reactivation of T-cell activity, while TNFSF agonists, such as OX40 and 4-1BB, further amplify T-cell activation and overcome the immunosuppressive TME, effectively transforming “cold” tumors into “hot” ones and bolstering immune responses in autoimmune diseases.

Despite the immense potential of this combined approach, several challenges remain, particularly with regard to irAEs, target specificity, and optimizing patient selection. Advances in precision medicine, including biomarker discovery and personalized diagnostics, will be key in identifying patients most likely to benefit from these therapies and minimizing toxicity. Additionally, next-generation TNFSF agonists and combination strategies are essential to further improve efficacy and safety. By integrating ICIs and TNFSF agonists with other therapeutic modalities and fostering interdisciplinary collaboration, this strategy holds significant promise in advancing immunotherapy and providing new hope for patients with both cancer and autoimmune diseases.
